# Ileocaecal recurrence of Merkel cell carcinoma of the skin: a case report

**DOI:** 10.1186/1752-1947-4-43

**Published:** 2010-02-08

**Authors:** Michelle Cheung, Henry Lee, Sanjay Purkayastha, Robert Goldin, Paul Ziprin

**Affiliations:** 1 Academic Surgical Unit, St Mary's Hospital, Imperial College Healthcare, Praed Street, London W2 1NY, UK

## Abstract

**Introduction:**

Merkel cell carcinoma is an uncommon skin malignancy that has a high propensity for metastatic spread. A systematic literature search identified 17 cases describing metastasis to the gastrointestinal tract, with 7 cases involving the small or large bowel. To the best of our knowledge, this is the only case described of Merkel cell carcinoma metastasising to the ileocaecal valve.

**Case presentation:**

We present a 74-year-old Filipino woman diagnosed with Merkel cell carcinoma of the skin with regional node involvement. Following excision and radiotherapy, the tumour recurred with metastasis to the ileocaecal valve. The patient died 28 months after the initial diagnosis.

**Conclusion:**

The prognosis of metastatic Merkel cell carcinoma is poor. Currently the optimal management for metastatic disease is unclear and lacks a firm evidence base due to the small number of cases reported.

## Introduction

Merkel cell carcinoma (MCC) is an uncommon and highly aggressive skin malignancy. It arises from Merkel cells at the dermo-epidermal junction, which are of neuroendocrine origin. Since its first description by Toker in 1972 [1], more than 2000 cases have been reported in the literature [2]. Its aetiology is not entirely known, but there is convincing evidence for the role of ultraviolet radiation. MCC has a predilection for sun-exposed areas of the body and is associated with other sun-related skin cancers such as basal cell carcinoma and squamous cell carcinoma. The occurrence of MCC in areas that are not exposed to the sun suggests additional causes. Reports of MCC in organ transplant, human immunodeficiency virus (HIV) infection and lymphohemopoietic malignancies, such as chronic lymphocytic leukemia, implicate a role for immunosuppression [2,3].

The incidence of MCC is 0.23 per 100,000 in Caucasians [2], which is about 20 times the incidence compared to the Afro-Caribbean population. MCC is also more common in older men with a mean age of diagnosis at 69 years old [2]. In a review of 1024 patients, the primary tumour was found in the head and neck in 40%, in the extremities in 33%, and in the trunk in 23% of patients [4]. At presentation the regional lymph nodes are involved in around 25% of cases, and distant metastases are found in 4% [4]. Metastasis usually occurs in the skin (28%), lymph nodes (27%), liver (13%), lung (10%), bone (10%), and the brain (6%) [2,4]. Metastasis can also involve the gastrointestinal (GI) tract in very rare cases.

A systematic search of the literature (see Appendix) found 17 cases involving GI metastases most commonly involving the stomach. Seven of these cases described bowel metastasis (Table 1). Shalhub *et al*. described the case of a 62-year-old man with axillary lymphadenopathy and metastasis to the stomach and the descending colon. The patient had a skin lesion excision which was initially diagnosed as basal cell carcinoma [5]. There are two case reports of stomach and small bowel MCC presenting with upper GI bleed from Krasagakis *et al*. [6] and Canales *et al*. [7]. An 85-year-old Japanese woman was also diagnosed with widespread upper GI tract MCC metastasis on autopsy following intestinal obstruction [8]. Naunton Morgan and Henderson reported a man with an enlarging nodule on his shin, who presented a month later with melaena and where a metastatic MCC lesion in the proximal jejunum was found on surgical exploration [9]. Meanwhile, Foster *et al*. also described a case of Merkel cell metastasizing to the small bowel after a protracted time course [10]. In addition, there are cases reported of metastasis to the rectum, the anal canal, and the pancreas [11-21].

**Table 1:** Reported cases of gastrointestinal metastases of Merkel call cancer.

Author(s)	Site of Metastasis
Li M and Liu C [11]	Stomach
Cubiella J, *et al*. [12]	Stomach
Idowu M, *et al*. [13]	Stomach
Wolov K, *et al*. [14]	Stomach
Krasagakis K, *et al*. [6]	Stomach, small bowel
Canales L, *et al*. [7]	Stomach, small bowel
Shalhub S, *et al*. [5]	Stomach, descending colon
Hizawa K, *et al*. [8]	Stomach, distal duodenum, pancreas
Olivero G, *et al*. [15]	Intestinal
Naunton M and Henderson RG [9]	Jejunum
Foster R, *et al*. [10]	Small bowel
Huang W S, *et al*. [16]	Rectum
Paterson C, *et al*. [17]	Anal canal
Adsay NV, *et al*. [18]	Pancreas
Bachmann J, *et al*. [19]	Pancreas
Dim DC, *et al*. [20]	Pancreas
Ouellett JR, *et al*. [21]	Pancreas

## Case presentation

A 74-year-old Filipino woman presented with a skin lesion in her right antecubital fossa. It was a 2 cm soft, mobile, well-circumscribed mass which appeared over three weeks. Suspecting liposarcoma, her general practitioner referred her to the hospital's surgical team. Urgent excision biopsy revealed metastatic carcinoma expressing neuroendocrine markers. Immunohistochemical staining showed that the patient had a strong positivity for CK20, which was a sensitive and specific marker for Merkel cell carcinoma [22]. She was also highly positive for chromogranin, synaptophysin and CD117, thus confirming the diagnosis of MCC. She showed no staining for TTF-1, which excluded lung primary small cell carcinoma.

Subsequent computed tomography (CT) examinations of her chest, abdomen and pelvis identified significant right-sided axillary lymphadenopathy, with the largest node measuring 6 cm in diameter. The CT was conducted two months after our patient's initial clinical presentation, and the axillary adenopathy was now clinically apparent. There was no hilar or mediastinal lymphadenopathy observed. Her lungs, bones and intra-abdominal organs were all clear of metastasis. A positron emission tomography (PET) body scan showed high uptake in her right axilla, with moderate heterogeneous uptake in some bowel loops. This was thought to represent inflammatory change in the presence of diverticular disease. No definite primary source, however, was identified.

At axillary lymph node dissection, eight of 26 lymph nodes, as well as the axillary vein, were found to contain tumour. Adjuvant radiotherapy was administered to our patient's axillary and subclavicular regions (42 Gy, 21 fractions, 31 days), but it was postponed until six months postoperatively due to the presence of seroma and lymphoedema. Surgical and oncological follow-up at intervals of two, three and six months found our patient asymptomatic.

**Figure 1:**
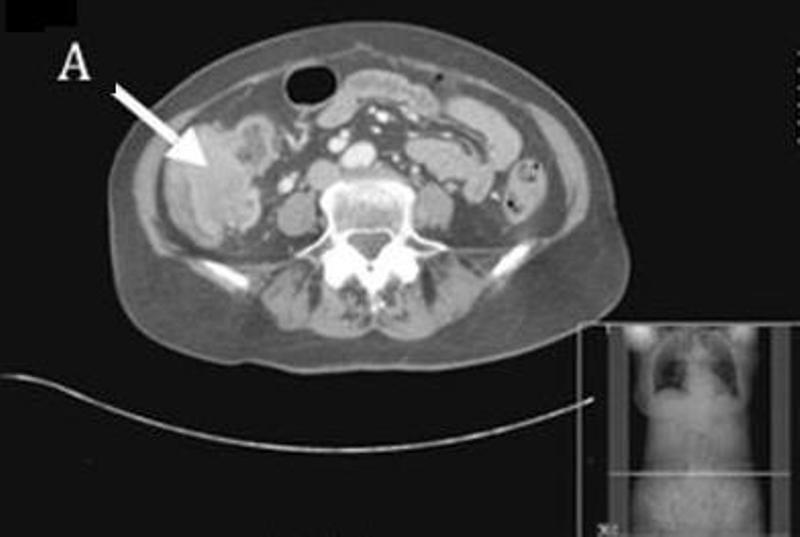
**Computed tomography showing a recurrence of Merkel cell carcinoma in the caecum at 18 months after the initial diagnosis**. There is a 6 × 4.3 cm soft-tissue mass seen in the region of the ileocecal valve (A) which extends into the lumen of the caecum. There are multiple abnormal lymph nodes measuring up to 2 cm within the ileocolic mesentery. There is nodularity and irregularity seen around the tumour extending into the pericolic fat suggestive of a local tumour infiltration.

**Figure 2:**
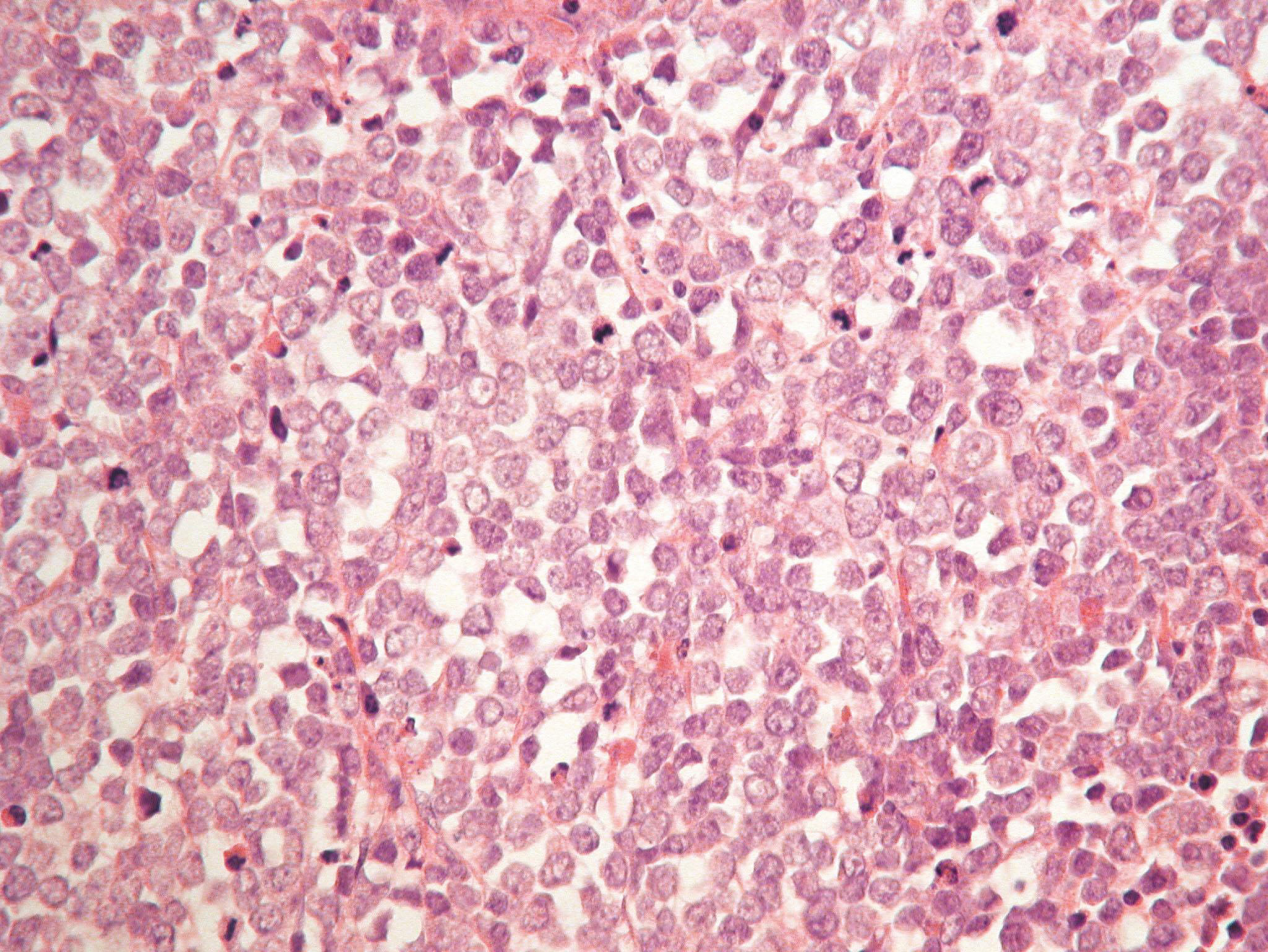
**Microscopic appearance of the ileocaecal valve tumour**. The mucosa shows infiltration by malignant cells with glassy nuclei, prominent nucleoli and scant cytoplasm. There is a high proliferation fraction. Immunostaining was positive for CK20, chromgranin A, synaptophysin, CD56, and MNF116. It was negative for CD3, CD30, CD10, CD20, CD23, CD5. The morphology and immunoprofile confirms this to be a metastatic Merkel cell carcinoma.

At 18 months from the initial diagnosis, our patient was referred by her general practitioner for urgent review with symptoms of obstruction, specifically early satiety, bloating, colicky pains and occasional vomiting. On examination, a firm mass in her right upper quadrant was clinically detectable. A whole body CT scan found a tumour in the ileocaecal valve that extended into the caecal lumen (Figure 1 and Figure 2). The tumour was associated with mesenteric lymphadenopathy and local infiltration into the pericolic fat and vessels.

At colonoscopy a tumour was seen at 70 cm from our patient's anus. The morphology and immunoprofile of the biopsy confirmed it as a recurrence of MCC. Markers for colorectal cancer (CA 19.9, CEA) were negative.

**Figure 3:**
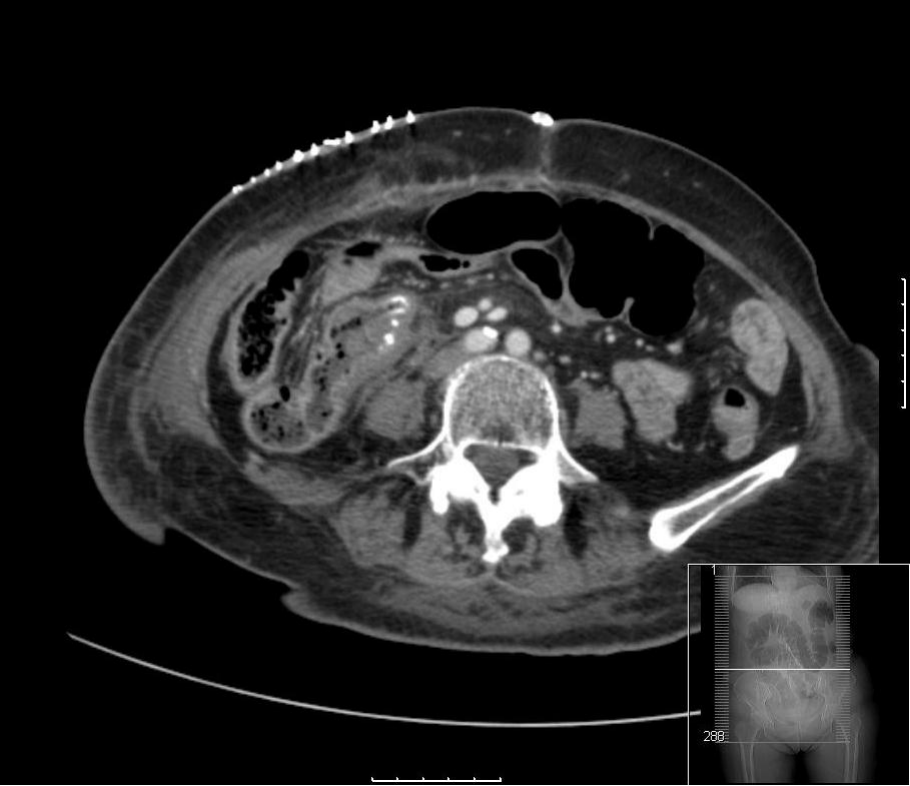
**Postoperative computed tomography scan following laparoscopic right hemicolectomy**. There is an end-to-side anastamosis between the distal ileum and the colon.

Our patient underwent a laparoscopic right hemicolectomy. At surgery the tumour was seen to be fungating and involved the full thickness of her bowel wall. Histological examination showed clear resection margins but vascular invasion and multiple lymph node involvement were also noted. Our patient's postoperative recovery was uneventful. Follow-up CT scan 3 months after the resection revealed peritoneal deposits with no recurrence in the right axilla (Figure 3). She was then scheduled for palliative chemotherapy.

Prior to chemotherapy our patient became increasingly frail and deteriorated rapidly. She died of bronchopneumonia 28 months after the initial diagnosis of metastatic Merkel cell carcinoma.

## Discussion

Merkel cell carcinoma has been described as one the most aggressive of the skin malignances [4,22]. In Medina-Franco's review of 1024 cases, this metastatic disease affects 31% of patients either at presentation or at later stage [2]. Excluding the patients with metastasis at presentation, 31% develop local recurrence, and the average disease-free interval is only 7.4 months [2]. Of the factors related to prognosis in MCC, lymph node status has been shown to be most consistent with clinical outcome [22-25]. This is reflected in the staging of the disease, where stage I describes lesions confined to the skin, stage II describes the involvement of regional nodes, and stage III describes the presence of metastasis. Five-year survival rates are 64%, 47% and 11%, respectively [2,23,25,26]. This compares with a 7% to 19% survival rate for malignant melanoma with metastasis [27].

A retrospective review of 109 patients by Allen *et al*. revealed that in patients with stage I disease, the tumour size at presentation was also an independent predictor of survival. In view of this, a 4-tiered system is also widely used to reflect a more accurate prognosis. It classifies patients with lymph node-negative cutaneous disease of <2 cm as stage I, and cutaneous lesions of >2 cm as stage II. Lymph node-positive disease is classified as stage III, while the presence of distant metastases is stage IV [25].

The treatment of choice for MCC is surgical excision with wide margins. There is no consensus on the width of excision required, but many surgical series have suggested margins of 2.5 cm to 3 cm [4]. There is evidence to show, however, that low local recurrence rates (8%) can be achieved after margin-negative excisions with margins that averaged 1.1 cm. This has lead to the suggestion that larger excision margins of at least 2 cm should be reserved for larger lesions of >2 cm in diameter [23].

Given that MCC occurs so frequently in the head and the neck, where wide local excision or even Moh's surgery may be difficult, the role of adjuvant therapy has been much considered. One large case series review involving over 400 patients found that radiotherapy improves local control from a recurrence rate of 52% to 10.5% [4]. Some studies, however, showed contrary results where radiotherapy did not improve recurrence rates in patients with node-negative diseases. They instead recommend that adjuvant therapy may be beneficial in patients with clinically positive nodes or those who have undergone elective or sentinel node biopsy with positive nodes [23].

Due to the importance of lymph node status in treatment outcome, sentinel lymph node biopsy (SLNB) as a staging procedure is considered standard practice in some centers, and its use has been recommended by many others. A number of studies have reported SLNB positivity rate of 20% to 30% involving patients who are clinically lymph node negative [23,28]. Where micrometastasis is found on SLNB, however, complete lymph node dissection is considered as first-line management, while radiotherapy is second-line [26,28].

As a neuroendocrine tumour, MCC demonstrates chemosensitivity to agents used in treating small-cell lung cancer, such as carboplatin, vincristine and etoposide. The role of chemotherapy, however, is much less established than that of radiotherapy. There is some support for its use either as a radiation sensitizer or as adjuvant therapy in high-risk cases with the disease at stage II or above [2]. At stage IV of the disease chemotherapy has shown high but relatively short response rates of 8 months on the average, and does not appear to add significantly where the median survival in metastasis is 10 months [28].

Overall, the optimal management for patients with MCC remains unclear. Treatment is often guided by opinion rather than evidence-based practice [2]. Bichakjian *et al*. proposed an algorithm reflecting the evidence as discussed above [28]. Again it suggests wide local excision margins of 1 cm for lesions measuring <2 cm, and 1 cm to 2 cm for lesions measuring >2 cm. Radiotherapy is best used when clear margins cannot be obtained. Furthermore, sentinel node biopsy should be performed in all instances.

Meanwhile, negative SLNB should be observed at intervals between 2 to 6 months for 2 years, with decreasing intervals thereafter. The use of imaging should be guided by clinical findings rather than by routine due to high false-positive rates, and the lack of survival benefit in detecting asymptomatic distant metastases. Positive sentinel nodes should be treated with complete node dissection and radiation depending on the level of the nodal disease. Once there is involvement in distant sites, therapy is primarily for palliation. The literature on the management of recurrent and metastatic diseases is particularly limited. The only consensus seems to be that the outcome of high stage MCC is gravely unfavorable.

## Conclusion

The management of Merkel cell carcinoma poses a challenge to clinicians for several reasons. For one, it is aggressive and has a propensity for spread and recurrence. Optimal therapy in high stage disease or recurrence is less clearly established. Its low incidence warrants the collaboration of different institutions to produce large multicenter controlled trials in order to compare responses to different treatment modalities. At any stage, its management is likely to benefit from a multidisciplinary approach, which is highlighted in this case report.

## Consent

Written informed consent was obtained from the patient's next-of-kin for publication of this case report and any accompanying images. A copy of the written consent is available for review by the Editor-in-Chief of this journal.

## Competing interests

The authors declare that they have no competing interests.

## Authors' contributions

MC reviewed the case notes and the literature. She also drafted the manuscript. HL and SP revised the references and finalized the manuscript. RG performed the histopathology examinations and provided the specimen image. PZ performed the patient's operation and conceived of the article. All authors read and approved the final manuscript.

## Appendix

Systematic search algorithm employed.

Literature search was conducted using MeSH terms "oesophagus" [All Fields] OR "esophagus" [All Fields] OR "stomach" [All Fields] OR "duodenum" [All Fields] OR "jejunum" [All Fields] OR "ileum" [All Fields] OR "bowel" [All Fields] OR "intestine" [All Fields] OR "intestinal" [All Fields] OR "caecum" [All Fields] OR "rectum" [All Fields] OR "rectal" [All Fields] OR "colon" [All Fields] AND "merkel cell" [All Fields].

This produced 61 results on PubMed of which 17 are reports of metastatic Merkel cell carcinoma in the gastrointestinal tract (excluding the liver).
